# Synthesis, Spectroscopic Properties and Antipathogenic Activity of New Thiourea Derivatives

**DOI:** 10.3390/molecules16097593

**Published:** 2011-09-06

**Authors:** Carmen Limban, Luminita Marutescu, Mariana Carmen Chifiriuc

**Affiliations:** 1 Pharmaceutical Chemistry Department, University of Medicine and Pharmacy Carol Davila, Bucharest, Romania; E-Mail: carmen_limban@yahoo.com; 2 Microbiology Department, Faculty of Biology, University of Bucharest, Ale. Portocalelor 1-4, Bucharest 60101, Romania; E-Mail: lumi_dascalu@yahoo.com; 3 National Institute for Research and Development in Microbiology and Immunology “Cantacuzino”, Spl. Independentei 103, Bucharest 060031, Romania

**Keywords:** acylthioureas, benzamides, 2-(4-methylphenoxymethyl)benzoic acid, antimicrobial activity, bacterial biofilm

## Abstract

A number of acylthioureas, 2-((4-methylphenoxy)methyl)-*N*-(aryl-carbamothioyl)benzamides (aryl = 3,5-dichlorophenyl, 2,3-dichlorophenyl, 3,4-dichloro-phenyl, 2,4,5-trichlorophenyl, 3,4,5-trichlorophenyl, 2-bromophenyl, 2,4-dibromophenyl, 2,5-dibromophenyl, 2-iodophenyl, 3-fluorophenyl, 2,3,4-trifluorophenyl, 2,4,5-trifluoro-phenyl, 2,4,6-trifluorophenyl) have been synthesized, characterized by elemental analysis, IR and NMR spectroscopy and tested for their interaction with bacterial cells in free and adherent state. The anti-pathogenic activity was correlated with the presence of one iodine, bromide or fluorine, and two or three chloride atoms on the N-phenyl substituent of the thiourea moiety, being significant especially on *Pseudomonas aeruginosa* and *Staphylococcus aureus* strains, known for their ability to grow in biofilms. Our results demonstrate the potential of these derivatives for further development of novel anti-microbial agents with antibiofilm properties.

## 1. Introduction 

Nowadays antibiotic resistance has become a serious public health problem. As a result of the rapid development of resistance to the existing portfolio of antimicrobial drugs, there is an increasing need to design new antibacterial and antifungal agents with better activity profiles and lower toxicity.

In recent years, thiourea derivatives have become a subject of interest due to their antibacterial, antifungal, antimycobacterial and antileprotic activities. A thiourea system attached to a polycyclic imide, such as thiourea derivatives of 4-azatricyclo[5.2.2.0^2,6^]undec-8-ene-3,5-dione, showed significant inhibitory activity against Gram-positive cocci [1]. Some pyridazine derivatives carrying thiourea moieties exhibit potent inhibitory activity against *Staphylococcus aureus*, *Escherichia coli*, *Candida albicans* and *C. parapsilosis* [2]. 1-Aroyl-3-arylthioureas showed moderate to potent activity against different bacterial strains, especially against *E. coli* strains resistant to standard drugs [3]. The antibacterial activity of the 1-aroyl-3-(substituted-2-benzothiazolyl)thioureas makes these substances promising agents for the treatment of infections. [4]. *N^1^,N^2^*-disubstituted thioureas, such as *N*-phenyl- and *N-*benzoylthioureas, have also attracted attention due to their potential application in medicinal chemistry as antimicrobial agents [5]. 

Novel aroyl, thiophenoyl, morpholinoyl and butanoyl thiourea derivatives containing a thiazole moiety, such as *N*-[(1,3-thiazol-2-ylamino)carbonothioyl]thiophene-2-carboxamide and *N*-[(1,3-thiazol-2-ylamino)carbonothioyl]morpholine-4-carboxamide exhibit broad spectrum antifungal activity [6]. For example, the 1-(isomeric fluorobenzoyl)-3-(isomeric fluorophenyl)thioureas were proven to exhibit better antifungal than antibacterial activity, probably due to the inclusion of fluorine that may increase the lipophilicity and enhance the rate of cell penetration and transport of the drug to an active site. The higher polarizability due to the C-F bond may provide new possibilities for binding to the receptor [7]. 

*N*-phenyl-*N′*-[4-(5-cyclohexylamino-1,3,4-thiadiazole-2-yl)phenyl]thiourea is active against *Mycobacterium tuberculosis* H_37_Rv [8]. In an effort to improve the therapeutic value of thiocarlide, several *N*-pentofuranosyl-*N*″-[*p*-isoamyloxy)phenyl]thioureas were tested as potential anti-tuberculosis therapeutic agents. *N*-d-Aldopentofuranosyl-*N′*-[p-(isoamyloxy)phenyl]thiourea derivatives, designed as structural analogues of thiocarlide, have recently been reported to be more potent than thiocarlide itself [9]. Novel 1-(5-cyclobutyl-1,3-oxazol-2-yl)-3(sub)phenyl/ pyridyl thiourea compounds inhibited *Mycobacterium tuberculosis* H_37_Rv and a clinical isolate of multidrug- resistant *M. tuberculosis* [10].

Many articles have presented the progress in the investigation of new thiourea derivatives as anthelmintics [11], antimalarials [12], antitumor agents [13], analgesics [14] and antivirals [15,16], these compounds proving also useful as insecticides [17,18], fungicides [19], herbicides [20], and plant-growth regulators [21]. Metals complexes with N-acyl thiourea ligands have been described in the literature with regard to their synthesis, general characterization and biological activity, such as cancerostatic activity [22]. 

Taking into account the improvement of the antimicrobial activity by the inclusion of fluorine in the organic molecule of thioureas [7], we synthesized, characterized and studied the antimicrobial activity of the new 2-((4-methylphenoxy)methyl)-*N*-(arylcarbamothioyl)benzamides, substituted with phenyl groups carrying one, two or three atoms of F, Cl, Br and I.

## 2. Results and Discussion 

### 2.1. Chemistry

Our decision to synthesize and evaluate this kind of substances for their antimicrobial activity was influenced by several reports disclosing the antimicrobial activities of thiourea-based compounds. We have previously synthesized and demonstrated the microbicidal activities of 2-(4-methoxyphenoxy-methyl)benzoic acid [23], 2-(4-chlorophenoxymethyl)benzoic acid [24], 2-(4-fluorophenoxymethyl)-benzoic acid [25], 2-(4-ethylphenoxymethyl)benzoic acid [26,27], and 2-(4-methylphenoxymethyl)-benzoic acid thioureides [28].

**Scheme 1:**
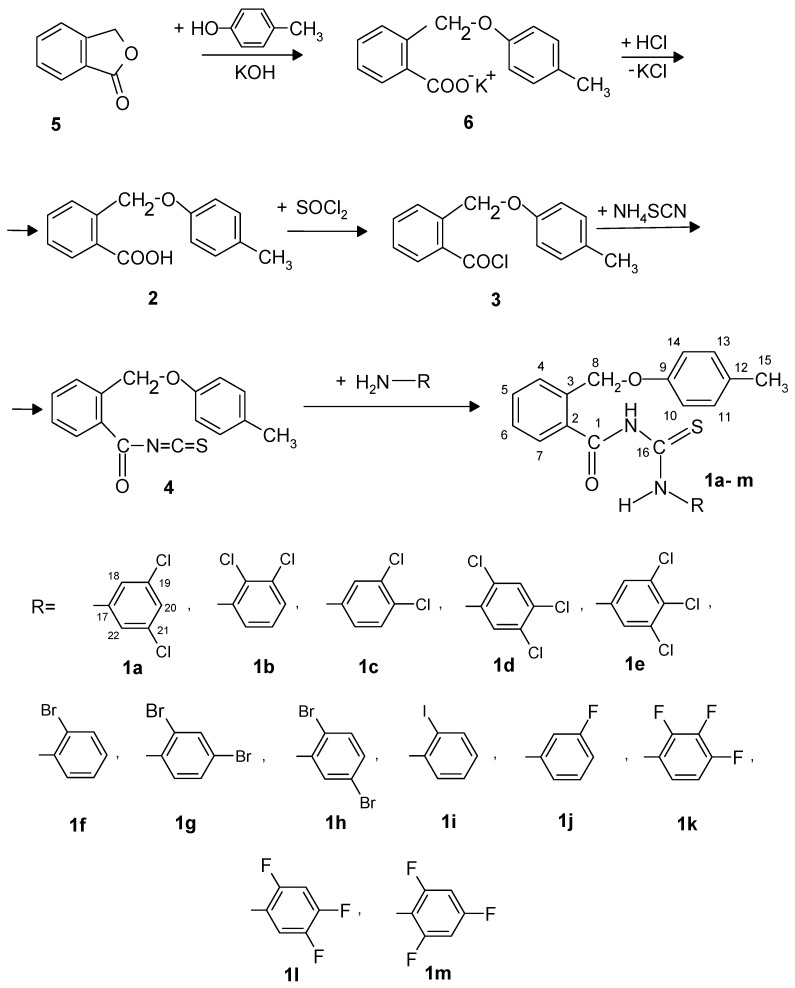
Synthesis of the new compounds.

The new derivatives **1a-m** were synthesized in good yields following the method described by us (Scheme 1). 2-(4-Methylphenoxymethyl)benzoic acid (**2**) was converted into the corresponding acid chloride **3** using thionyl chloride and anhydrous 1,2-dichloroethane as reaction medium, then treated with a solution of ammonium thiocyanate in acetone to afford arylisothiocyanate **4** *in situ*, followed by refluxing with amines to provide acylthioureas **1a-m** in good yields. The compounds were purified by recrystallization from isopropanol. The acid **2** was synthesized with the best yield by using phtalide **5**, which was treated with potassium *para*-cresolate in xylene under reflux. The obtained potassium salt **6** of 2-(4-methylphenoxymethyl)benzoic acid, due to its good solubility in 10% aqueous potassium hydroxide solution allowed its facile separation from xylene. The acid **2** was then precipitated using a mineral acid solution. The necessary potassium *para*-cresolate was obtained from the corresponding phenol and potassium hydroxide in xylene. The resulting water was removed by azeotropic distillation.

The new compounds have all been characterized by their melting point, elemental analysis, infrared and NMR spectral studies. All spectroscopic and elemental analyses data confirm the proposed structures of the new compounds.

### 2.2. Biological Activity

#### 2.2.1. Antimicrobial Activity

The emergence of bacterial resistance and multiresistance to antibiotics has been declared by the European Center for Disease Control (ECDC) as one of the major public health problems, besides HIV infection, tuberculosis and influenza. The increasing occurrence of multiresistant pathogenic bacterial strains has gradually rendered traditional antimicrobial treatment ineffective. Consequently, there is a pressing need to develop new antibiotics in order to overcome the bacterial resistance [29]. 

The qualitative screening of the susceptibility spectra of various microbial strains to the newly synthesized compounds showed that only five of the tested compounds (**1c, 1d, 1f, 1i** and **1j**) exhibited an antimicrobial effect quantified by the occurrence of a growth inhibition zone (Table 1). The compounds **1c** (substituted with two chloride atoms in positions 3 and 4) and **1j** (containing one fluorine atom in position 3) exhibited activity against *S. aureus* ATCC 25923 strain, while **1i** (containing one iodine atom in position 2) exhibited activity against *S. aureus* 1694 clinical strain. The compound **1d** (substituted with three chloride atoms in positions 2, 4 and 5) exhibited anti-*Pseudomonas* activity, and **1f** (containing one bromide atom in position 2) was active on *P. aeruginosa* and *K. pneumoniae* strains. The compound **1j** was the only one active both on Gram negative *P. aeruginosa* and the Gram positive *S. aureus*. 

The results of the quantitative assay revealed that the serial dilutions of the compounds that proved to be active on different microbial strains in the qualitative screening assay, exhibited a low inhibitory activity on the bacterial growth, with high MIC values of 1,000–500 μg/mL (Table 2). Despite the high MIC values obtained for the tested compounds, we further tested them for their anti-pathogenic features, taking into account that, in many cases, the sub-inhibitory concentrations of the antimicrobial substances interfere with the expression of different virulence features, such are adhesins or toxins [30]. 

#### 2.2.2. Antipathogenic Activity

The genetic resistance of different microbial strains to numerous antimicrobial agents is amplified when one organism is found growing in a biofilm. Antimicrobial resistance is a trait typical of most biofilm organisms and it has been speculated that biofilms are the causative agent of up to 65% of bacterial infections [31]. Biofilms are thought to become recalcitrant to antimicrobial assault through a number of different mechanisms. 

Giving the increasing risk of antimicrobial resistance, research is focusing on the discovery of anti-pathogenic drugs that block the coordinated expression of virulence factors, such as adherence capacity or production of toxins, thus rendering the bacteria harmless. Contrary to antibiotics, these new anti-pathogenic drugs do not kill the bacteria, and since the survival of the bacteria is not threatened by this approach, the development of resistance, like that to antibiotics, is not anticipated to be a serious problem. 

The inert substrata including the prosthetic medical devices represent risk factors for the occurrence of biofilm associated infections. Taking into account the differences in physiology and susceptibility to antibiotics of biofilm-embedded bacteria [32], the compounds active against different bacterial strains in free state were further chosen and investigated concerning their efficiency against the adherent cells grown in biofilms developed in plastic wells. The experimental model uses mini volumes and multiple well plastic plates, allowing the simultaneous testing of a large spectrum of antibiotic concentrations. 

Our results showed that the five compounds selected for the quantitative MIC assay proved to be extremely active, inhibiting the ability of the *S. aureus, Ps. aeruginosa* and *K. pneumoniae* strains to adhere and colonize the inert substratum at very low concentrations (with one exception, ranging between 13 µg/mL and 27 µg/mL), probably due to their interference with adhesins expression (Table 3). Thus, despite their high MICs, the tested substances seem to be promising as antipathogenic compounds, which may be used in low concentrations for prophylaxis or therapy by themselves or in combination with an antibiotic.

## 3. Experimental 

### 3.1. General

All chemicals used for the preparation of the compounds were of reagent grade quality and were obtained from Merck, Fluka or Sigma- Aldrich. *p*-Methylphenol was used freshly distilled. Acetone was dried over K_2_CO_3_ and then distilled and ammonium thiocyanate was treated by heating at 100 °C before use. The necessary liquid amines were dried with potassium hydroxide and afterwards distilled.

Melting points were determined in open capillary tubes on an Electrothermal 9100 apparatus and uncorrected. C, H, N and S analysis were carried out on a Perkin Elmer CHNS/O Analyzer Series II 2400 elemental analyzer. The room temperature attenuated total reflection Fourier transform infrared (FT-IR ATR) spectra of the all synthesized compounds were registered using a Bruker Vertex 70 spectrophotometer. The IR bands are given as w – weak, m – medium, s – intense, vs – very intense. ^1^H-NMR and ^13^C-NMR spectra were recorded in DMSO-d_6_ on a Gemini 300BB instrument, at room temperature, operating at 300 MHz for ^1^H and 75 MHz for ^13^C and a Unity Inova 400 instrument, operating at 400 MHz for ^1^H and 100 MHz for ^13^C. Chemical shifts were given as δ values in parts per million (ppm) relative to tetramethylsilane as internal standard. Coupling constants J were given in Hz. Spin multiplets are given as s (singlet), d (doublet), t (triplet), q (quartet), m (multiplet), dd (double doublet), td (triple doublet) and br (broad). The chemical shifts for hydrogen and carbon atoms were established also by G-COSY, G-HSQC, G-HMBC experiments. The 2-(4-methylphenoxymethyl)-benzoic acid and 2-(4-methyl-phenoxymethyl)benzoyl chloride derivatives were obtained in good yields similar to those reported in the previous article [33].

#### 3.1.1. General Synthesis Procedure of the New Thioureides

A solution of 2-(4-methylphenoxymethyl)benzoyl chloride (0.01 mol) in acetone (15 mL) was added to a solution of ammonium thiocyanate (0.01 mol) in acetone (5 mL). The reaction mixture was heated under reflux for 1 h, and then cooled at the room temperature. A solution of primary amine (0.01 mol) in acetone (2 mL) was added to the mixture and heated under reflux for 1 h. The thioureide was precipitated after the cooled reaction mixture was poured into 500 mL water. The solid product was purified by recrystallization from isopropanol with active carbon.

#### 3.1.2. Spectral Data

*2-((4-Methylphenoxy)methyl)-N-(3,5-dichlorophenylcarbamothioyl)benzamide* (**1a**); yield 70%, mp 197.1–198.9 °C. ^1^H-NMR: 12.31 (s, 1H, NH, D_2_O exchangeable); 11.98 (br s, 1H, NH, D_2_O exchangeable); 7.67 (d, *J =* 1.7 Hz, 2H, H-18, H-22); 7.61 (bd, *J =* 7.4 Hz, 1H, H-7); 7.58 (dd, *J =* 1.6 Hz, *J =* 7.4 Hz, 1H, H-4); 7.55 (td, *J =* 1.4 Hz, *J =* 7.4 Hz, 1H, H-5); 7.50 (t, *J =* 1.7 Hz, 1H, H-20); 7.46 (td, *J =* 1.4 Hz, *J =* 7.8 Hz, 1H, H-6); 7.05 (d, *J =* 8.6 Hz, 2H, H-11, H-13); 6.87 (d, *J =* 8.6 Hz, 2H, H-10, H-14); 5.26 (s, 2H, H-8); 2.22 (s, 3H, H-15); ^13^C-NMR: 179.34 (C-16); 169.92 (C-1); 156.05 (C-9); 140.19 (C-3); 135.80 (C-19, C-21); 133.52 (C-2); 133.18 (C-12); 130.98 (C-5); 129.69 (C-11, C-13); 129.58 (C-17); 128.35 (C-4); 128.23 (C-7); 127.68 (C-6); 125.51 (C-20); 123.07 (C-18, C-22); 114.42 (C-10, C-14); 67.41 (C-8); 19.99 (C-15); FT-IR (ν cm^−1^): 3278m; 3075w; 3018w; 2914w; 2857w; 1675m; 1621w; 1575m; 1543s; 1513vs; 1440m; 1386m; 1315m; 1292m; 1243s; 1160s; 1107w; 1042m; 988w; 885w; 854w; 815w; 798w; 752m; 689m; 651w; 607w; Anal. Calcd for C_22_H_18_Cl_2_N_2_O_2_S (445.36): C, 59.33; H, 4.07; N, 6.29; S, 7.20%; Found: C, 59.65; H, 4.23; N, 6.34; S 7.25%.

*2-((4-Methylphenoxy)methyl)-N-(2,3-dichlorophenylcarbamothioyl)benzamide* (**1b**); yield 65%, mp 152.4–153.2 °C; ^1^H-NMR: 12.43 (s, 1H, NH, D_2_O exchangeable); 12.12 (br s, 1H, NH, D_2_O exchangeable); 7.87 (dd, *J =* 1.2 Hz, *J =* 8.2 Hz, 1H, H-22); 7.64 (dd, *J =* 1.2 Hz, *J =* 8.2 Hz, 1H, H-20); 7.61 (bd, *J =* 7.4 Hz, 1H, H-7); 7.58 (dd, *J =* 1.6 Hz, *J =* 7.4 Hz, 1H, H-4); 7.55 (td, *J =* 1.4 Hz, *J =* 7.4 Hz, 1H, H-5); 7.46 (td, *J =* 1.4 Hz, *J =* 7.8 Hz, 1H, H-6); 7.41 (t, *J =* 8.2 Hz, 1H, H-21); 7.05 (d, *J =* 8.6 Hz, 2H, H-11, H-13); 6.88 (d, *J =* 8.6 Hz, 2H, H-10, H-14); 5.27 (s, 2H, H-8); 2.21 (s, 3H, H-15); ^13^C-NMR: 180.15 (C-16); 170.17 (C-1); 156.02 (C-9); 137.35 (C-3); 135.77 (C-2); 133.13 (C-12); 131.77 (C-19); 129.61 (C-17); 127.33 (C-18); 131.02 (C-5); 129.69 (C-11, C-13); 128.51 (C-20); 128.48 (C-4); 128.40 (C-7); 127.73 (C-21); 127.64 (C-6); 126.80 (C-22); 114.51 (C-10, C-14); 67.51 (C-8); 19.99 (C-15); FT-IR (ν cm^−1^): 3252w; 3120w; 3026w; 2912w; 2855w; 1680m; 1573m; 1510vs; 1444m; 1418m; 1381m; 1318m; 1294m; 1240s; 1197m; 1163s; 1124m; 1108m; 1038m; 968w; 873w; 856w; 818w; 804w; 777w; 747m; 727m; 696m; 658m; 607w; 571w; Anal. Calcd for C_22_H_18_Cl_2_N_2_O_2_S (445.36): C, 59.33; H, 4.07; N, 6.29; S, 7.20%; Found: C, 59.68; H, 4.15; N, 6.24; S 7.37%.

*2-((4-Methylphenoxy)methyl)-N-(3,4-dichlorophenylcarbamothioyl)benzamide* (**1c**); yield 55%, mp 172.1–174.3 °C; ^1^H-NMR: 12.33 (s, 1H, NH, D_2_O exchangeable); 11.98 (br s, 1H, NH, D_2_O exchangeable); 7.97 (d, *J =* 2.3 Hz, 1H, H-18); 7.65 (d, *J =* 8.8 Hz, 1H, H-21); 7.54 (dd, *J =* 2.3 Hz, *J =* 8.8 Hz, 1H, H-22); 7.61 (bd, *J =* 7.4 Hz, 1H, H-7); 7.58 (dd, *J =* 1.6 Hz, *J =* 7.4 Hz, 1H, H-4); 7.57 (dd, *J =* 1.6 Hz, *J =* 8.2 Hz, 1H, H-22); 7.55 (td, *J =* 1.4 Hz, *J =* 7.4 Hz, 1H, H-5); 7.46 (td, *J =* 1.4 Hz, *J =* 7.8 Hz, 1H, H-6); 7.05 (d, *J =* 8.6 Hz, 2H, H-11, H-13); 6.87 (d, *J =* 8.6 Hz, 2H, H-10, H-14); 5.26 (s, 2H, H-8); 2.21 (s, 3H, H-15); ^13^C-NMR: 179.18 (C-16); 169.90 (C-1); 156.04 (C-9); 137.88 (C-18); 135.80 (C-2); 133.16 (C-12); 130.58 (C-19); 130.98 (C-5); 130.29 (C-22); 129.70 (C-11, C-13); 129.59 (C-17); 128.39 (C-4); 128.26 (C-7); 128.07 (C-20); 127.65 (C-6); 125.92 (C-18); 124.73 (C-21); 114.46 (C-10, C-14); 67.40 (C-8); 19.98 (C-15); FT-IR (ν cm^-1^): 3330m; 2969m; 1679m; 1601m; 1584m; 1505vs; 1469s; 1443s; 1384m; 1332m; 1293m; 1227s; 1173s; 1144s; 1050w; 1026s; 971w; 873w; 853w;821m; 787w; 749m; 709m; 664m; 608w; 567w; Anal. Calcd for C_22_H_18_Cl_2_N_2_O_2_S (445.36): C, 59.33; H, 4.07; N, 6.29; S, 7.20%; Found: C, 59.76; H, 3.96; N, 6.21; S 7.23%.

*2-((4-Methylphenoxy)methyl)-N-(2,4,5-trichlorophenylcarbamothioyl)benzamide* (**1d**); yield 58%, mp 171.8–173.4 °C; ^1^H-NMR: 12.47 (br s, 1H, NH); 12.20 (br s, 1H, NH); 8.28 (s, 1H, H-22); 7.97 (s, 1H, H-19); 7.62 (bd, *J =* 7.4 Hz, 1H, H-7); 7.58 (dd, *J =* 1.6 Hz, *J =* 7.4 Hz, 1H, H-4); 7.55 (td, *J =* 1.4 Hz, *J =* 7.4 Hz, 1H, H-5); 7.46 (td, *J =* 1.4 Hz, *J =* 7.5 Hz, 1H, H-6); 7.04 (d, *J =* 8.6 Hz, 2H, H-11, H-13); 6.86 (d, *J =* 8.62 Hz, H, H-10, H-14); 5.26 (s, 2H, H-8); 2.21 (s, 3H, H-15); ^13^C-NMR: 179.89 (C-16); 170.23 (C-1); 156.01 (C-9); 135.75 (C-3); 135.49 (C-2); 133.11 (C-12); 131.05 (C-5); 130.40 (C-19); 129.66 (C-11, C-13); 129.58 (C-20); 129.32 (C-17); 129.31 (C-7); 128.71 (C-21); 128.48 (C-4); 128.27 (C-22); 127.87 (C-18); 127.77 (C-6); 114.44 (C-10, C-14); 67.51 (C-8); 19.99 (C-15); FT-IR (ν cm^−1^): 3553w; 3094w; 2989w; 2917w; 1678m; 1603w; 1566s; 1511vs; 1462m; 1446m; 1391w; 1364w; 1311m; 1260m; 1242s; 1165s; 1127w; 1078w; 1070w; 1052m; 1040m; 895w; 878w; 857w; 835w; 814w; 799m; 753m; 699m; 668w; 613w; 564w. Anal. Calcd for C_22_H_17_Cl_3_N_2_O_2_S (479.80): C, 55.04; H, 3.57; N, 5.84; S, 6.68%; Found: C, 55.31; H, 3.59; N, 5.71; S 6.60%.

*2-((4-Methylphenoxy)methyl)-N-(3,4,5-trichlorophenylcarbamothioyl)benzamide* (**1e**); yield 67%, mp 158.7–160.8 °C. ^1^H-NMR: 12.16 (br s, 2H, NH); 7.90 (s, 2H, H-18, H-22); 7.62 (bd, *J =* 7.4 Hz, 1H, H-7); 7.58 (dd, *J =* 1.6 Hz, *J =* 7.4 Hz, 1H, H-4); 7.55 (td, *J =* 1.4 Hz, *J =* 7.4 Hz, 1H, H-5); 7.46 (td, *J =* 1.4 Hz, *J =* 7.5 Hz, 1H, H-6); 7.05 (d, *J =* 8.6 Hz, 2H, H-11, H-13); 6.87 (d, *J =* 8.6 Hz, 2H, H-10, H-14); 5.26 (s, 2H, H-8); 2.21 (s, 3H, H-15). ^13^C-NMR: 179.51 (C-16); 169.87 (C-1); 156.04 (C-9); 137.90 (C-3); 135.82 (C-2); 133.10 (C-12); 132.32 (C-19, C-21); 129.59 (C-17); 126.79 (C-20); 131.03 (C-5); 129.69 (C-11, C-13); 128.36 (C-7); 128.29 (C-4); 127.69 (C-6); 125.06 (C-18, C-22); 114.42 (C-10, C-14); 67.40 (C-8); 20.00 (C-15). FT-IR (ν cm^−1^): 3290w; 3079w; 2912w; 1682m; 1588w; 1561m; 1536m; 1509vs; 1431m; 1381m; 1309m; 1248s; 1238s; 1992w; 1169s; 1150m; 1053w; 1039m; 887w; 860w; 813w; 804w; 782w; 752m; 718w; 688w; 666m; 611w. Anal. Calcd for C_22_H_17_Cl_3_N_2_O_2_S (479.80): C, 55.04; H, 3.57; N, 5.84; S, 6.68%; Found: C, 55.29; H, 3.52; N, 5.91; S 6.76%.

*2-((4-Methylphenoxy)methyl)-N-(2-bromophenylcarbamothioyl)benzamide* (**1f**); yield 62%, mp 144.5–145.7 °C. ^1^H-NMR: 12.38 (br s, 1H, NH); 12.02 (br s, 1H, NH); 7.85 (dd, *J =* 1.5 Hz, *J =* 8.1 Hz, 1H, H-22); 7.71 (dd, *J =* 1.5 Hz, *J =* 8.1 Hz, 1H, H-19); 7.62 (dd, *J =* 1.7 Hz, *J =* 6.6 Hz, 1H, H-7); 7.58 (dd, *J =* 2.4 Hz, *J =* 7.4 Hz, 1H, H-4); 7.55 (td, *J =* 1.7 Hz, *J =* 8.9 Hz, 1H, H-5); 7.47 (ddd, *J =* 2.4 Hz, *J =* 6.6 Hz, *J =* 8.9 Hz, 1H, H-6); 7.43 (ddd, *J =* 1.5 Hz, *J =* 7.3 Hz, *J =* 8.1 Hz, 1H, H-21); 7.24 (ddd, *J =* 1.5 Hz, *J =* 7.3 Hz, *J =* 8.1 Hz, 1H, H-20); 7.06 (d, *J =* 8.6 Hz, 2H, H-11, H-13); 6.88 (d, *J =* 8.6 Hz, 2H, H-10, H-14); 5.27 (s, 2H, H-8); 2.21 (br s, 3H, H-15). The doublet signal of the protons H-11 and H-13 in the ^1^H-NMR spectrum is further split by the methyl group. Scalar coupling is experimentally measured and the value has ^4^*J*(H11,H13-CH3)=0.6 Hz. ^13^C-NMR: 179.99 (C-16); 170.17 (C-1); 156.01 (C-9); 136.60 (C-3); 135.70 (C-2); 133.19 (C-12); 129.63 (C-17); 118.96 (C-18); 135.70 (C-19); 132.53 (C-20); 130.99 (C-5); 129.69 (C-11, C-13); 128.51 (C-7); 128.49 (C-22); 128.42 (C-21); 127.73 (C-6); 127.64 (C-20); 114.52 (C-10, C-14); 67.45 (C-8); 19.99 (C-15). FT-IR (ν cm^−1^): 3232w; 3027w; 2918w; 2857w; 1675m; 1578w; 1513vs; 1441m; 1387w; 1317w; 1287w; 1242s; 1161s; 1042m; 853w; 801w; 742m; 707m; 672w; 608w. Anal. Calcd for C_22_H_19_BrN_2_O_2_S (455.36): C, 58.02; H, 4.21; N, 6.15; S, 7.04%; Found: C, 57.89; H, 4.28; N, 6.11; S 7.16%.

*2-((4-Methylphenoxy)methyl)-N-(2,4-dibromophenylcarbamothioyl)benzamide* (**1g**); yield 67%, mp 169.3–170.6 °C. ^1^H-NMR: 12.33 (br s, 1H, NH); 12.10 (br s, 1H, NH); 7.95 (d, *J =* 1.4 Hz, 1H, H-19); 7.80 (d, *J =* 8.6 Hz, 1H, H-22); 7.64 (dd, *J =* 1.4 Hz, *J =* 8.6 Hz, 1H, H-21); 7.62 (bd, *J =* 7.4 Hz, 1H, H-7); 7.58 (dd, *J =* 1.6 Hz, *J =* 7.4 Hz, 1H, H-4); 7.55 (td, *J =* 1.4 Hz, *J =* 7.4 Hz, 1H, H-5); 7.46 (td, *J =* 1.4 Hz, *J =* 7.5 Hz, 1H, H-6); 7.05 (d, *J =* 8.6 Hz, 2H, H-11, H-13); 6.87 (d, *J =* 8.6 Hz, 2H, H-10, H-14); 5.26 (s, 2H, H-8); 2.21 (br s, 3H, H-15). ^13^C-NMR: 180.00 (C-16); 170.12 (C-1); 155.99 (C-9); 136.36 (C-3); 135.74 (C-2); 133.11 (C-12); 129.58 (C-17); 120.22 (C-20); 119.64 (C-18); 134.41 (C-19); 131.03 (C-5); 130.64 (C-21); 129.82 (C-22); 129.69 (C-4); 129.69 (C-11, C-13); 128.49 (C-7); 127.72 (C-6); 114.51 (C-10, C-14); 67.43 (C-8); 19.99 (C-15). FT-IR (ν cm^−1^): 3261w; 3130w; 3032w; 2913w; 2855w; 1679m; 1615w; 1560m; 1512vs; 1443w; 1376w; 1295w; 1246s; 1163s; 1078w; 1038m; 972w; 942w; 860w; 801w; 781w; 755w; 672m; 601w; 541w. Anal. Calcd for C_22_H_18_Br_2_N_2_O_2_S (534.26): C, 49.46; H, 3.40; N, 5.24; S, 6.00%; Found: C, 49.79; H, 3.29; N, 5.33; S 5.89%.

*2-((4-Methylphenoxy)methyl)-N-(2,5-dibromophenylcarbamothioyl)benzamide* (**1h**); yield 69%, mp 160.2–161.5 °C. ^1^H-NMR: 12.39 (br s, 1H, NH); 12.12 (br s, 1H, NH); 8.04 (d, *J =* 2.1 Hz, 1H, H-22); 7.66 (d, *J =* 8.5 Hz, 1H, H-19); 7.62 (bd, *J =* 7.4 Hz, 1H, H-7); 7.58 (dd, *J =* 1.6 Hz, *J =* 7.4 Hz, 1H, H-4); 7.55 (td, *J =* 1.4 Hz, *J =* 7.4 Hz, 1H, H-5); 7.46 (td, *J =* 1.4 Hz, *J =* 7.5 Hz, 1H, H-6); 7.43 (dd, *J =* 2.1 Hz, *J =* 8.5 Hz, 1H, H-20); 7.05 (d, *J =* 8.6 Hz, 2H, H-11, H-13); 6.87 (d, *J =* 8.6 Hz, 2H, H-10, H-14); 5.26 (s, 2H, H-8); 2.20 (br s, 3H, H-15). ^13^C-NMR: 179.93 (C-16); 170.15 (C-1); 156.00 (C-9); 138.18 (C-3); 135.68 (C-2); 133.21 (C-12); 129.57 (C-17); 119.60 (C-18); 118.25 (C-21); 134.09 (C-19); 131.00 (C-5); 130.95 (C-20); 130.61 (C-22); 129.67 (C-11, C-13); 128.50 (C-4); 128.43 (C-7); 127.77 (C-6); 114.44 (C-10, C-14); 67.50 (C-8); 20.03 (C-15). FT-IR (ν cm^−1^): 3241m; 2997w; 2916w; 1675m; 1567m; 1513vs; 1395m; 1313m; 1240s; 1160s; 1075w; 1043m; 957w; 874w; 814w; 790m; 754m; 700m; 608w; 562w. Anal. Calcd for C_22_H_18_Br_2_N_2_O_2_S (534.26): C, 49.46; H, 3.40; N, 5.24; S, 6.00%; Found: C, 49.21; H, 3.28; N, 5.15; S 6.07%.

*2-((4-Methylphenoxy)methyl)-N-(2-iodophenylcarbamothioyl)benzamide* (**1i**); yield 94%, mp 134.4–136.2 °C. ^1^H-NMR: 12.22 (br s, 1H, NH); 12.00 (br s, 1H, NH); 7.92 (dd, *J =* 1.4 Hz, *J =* 7.9 Hz, 1H, H-19); 7.65-7.55(m, 4H, H-4, H-5, H-7, H-22); 7.46 (td, *J =* 1.4 Hz, *J =* 7.5 Hz, 1H, H-6); 7.44 (td, *J =* 7.9 Hz, 1H, H-21); 7.07 (dd, *J =* 1.5 Hz, *J =* 7.9 Hz, 1H, H-20); 7.05 (d, *J =* 8.6 Hz, 2H, H-11, H-13); 6.87 (d, *J =* 8.6 Hz, 2H, H-10, H-14,); 5.27 (s, 2H, H-8); 2.22 (s, 3H, H-15). ^13^C-NMR: 180.13 (C-16); 170.07 (C-1); 156.01 (C-9); 140.06 (C-17); 139.77 (C-19); 135.69 (C-3); 133.19 (C-2); 131.02 (C-5); 129.72 (C-11, C-13); 129.56 (C-12); 128.74 (C-22); 128.57 (C-20); 128.52 (C-4); 128.45 (C-7); 128.36 (C-21); 127.72 (C-6); 114.56 (C-10, C-14); 96.89 (C-18); 67.35 (C-8); 19.98 (C-15). FT-IR (ν cm^−1^): 3389s; 3153w; 3029w; 2924w; 1684m; 1608w; 1511vs; 1485vs; 1379m; 1354m; 1308m; 1237s; 1142s; 1068w; 1019m; 877w; 795w; 743m; 716w; 653m; 595w; 515w. Anal. Calcd for C_22_H_19_IN_2_O_2_S (502.36): C, 52.60; H, 3.81; N, 5.58; S, 6.38%; Found: C, 52.86; H, 3.97; N, 5.43; S 6.34%.

*2-((4-Methylphenoxy)methyl)-N-(3-fluorophenylcarbamothioyl)benzamide* (**1j**); yield 76%, mp 116.9–119 °C. ^1^H-NMR: 12.48 (br s, 1H, NH); 11.89 (br s, 1H, NH); 7.69 (bd, ^3^*J*_(F-H)_= 10.9 Hz, 1H, H-18); 7.62 (bd, *J =* 7.4 Hz, 1H, H-7); 7.58 (dd, *J =* 1.6 Hz, *J =* 7.4 Hz, 1H, H-4); 7.55 (td, *J =* 1.4 Hz, *J =* 7.4 Hz, 1H, H-5); 7.46 (td, *J =* 1.4 Hz, *J =* 7.5 Hz, 1H, H-6); 7.43 (dd, ^3^*J*_(H-22-H-21)_= 8.2 Hz, ^3^*J*_(H-20-H-21)_= 8.6 Hz, 1H, H-21); 7.34 (ddd, *J =* 1.1 Hz, *J =* 1.9 Hz, *J =* 8.2 Hz, 1H, H-22); 7.10 (tdd, ^3^*J*_(F-H)_= 8.6 Hz, ^3^*J*_(H-20-H-21)_= 8.6 Hz, ^3^*J*_(H 20-H 21)_= 1.9 Hz, ^3^*J*_(H-20-H-18)_= 1.4 Hz, 1H, H-20); 7.05 (d, *J =* 8.6 Hz, 2H, H-11, H-13); 6.87 (d, *J =* 8.6 Hz, 2H, H-10, H-14); 5.27 (s, 2H, H-8); 2.20 (s, 3H, H-15). ^13^C-NMR: 178.91 (C-16); 170.00 (C-1); 161.57 (d, *J*_(F-C)_= 241.0 Hz, C-19); 156.05 (C-9); 139.48 (C-3); 135.81 (C-2); 133.16 (C-12); 129.60 (C-17); 130.98 (C-5); 130.19 (C-21); 129.71 (C-11, C-13); 128.43 (C-7); 128.27 (C-4); 127.64 (C-6); 120.05 (C-22); 114.49 (C-10, C-14); 112.80 (d, *J*_(F-C)_= 21.2 Hz, C-20); 110.95 (d, *J*_(F-C)_= 25.7 Hz, C-18); 67.40 (C-8); 19.96 (C-15). FT-IR (ν cm^−1^): 3345w; 3129m; 2921w; 2857w; 1675m; 1601m; 1551s; 1504vs; 1445s; 1380w; 1331m; 1302m; 1283m; 1271m; 1256m; 1227vs; 1176m; 1140s; 1056w; 1024m; 985w; 958w; 899w; 860w; 829w; 800w; 781w; 727m; 669m; 605w; 555w; 512w. Anal. Calcd for C_22_H_19_FN_2_O_2_S (394.46): C, 66.99; H, 4.85; N, 7.10; S, 8.13%; Found: C, 67.33; H, 4.72; N, 7.03; S 8.15%.

*2-((4-Methylphenoxy)methyl)-N-(2,3,4-trifluorophenylcarbamothioyl)benzamide* (**1k**); yield 71%, mp 111.2–112.3 °C. ^1^H-NMR: 12.13 (br s, 1H, NH); 12.10 (br s, 1H, NH); 7.63 (bd, *J =* 7.4 Hz, 1H, H-7); 7.62- 7.54 (m, 3H, H-4, H-5, H-22); 7.47 (td, *J =* 1.4 Hz, *J =* 7.5 Hz, 1H, H-6); 7.36 (ddd, *J*_(F-20-H-21)_= 9.5 Hz, ^3^*J*_(H-22-H-21)_= 8.2 Hz, ^4^*J*_(H-22-H-21)_= 2.3 Hz, 1H, H-21); 7.04 (d, *J =* 8.6 Hz, 2H, H-11, H-13); 6.86 (d, *J =* 8.6 Hz, 2H, H-10, H-14); 5.27 (s, 2H, H-8); 2.22 (s, 3H, H-15). ^13^C-NMR: 179.89 (C-16); 170.23 (C-1); 156.01 (C-9); 135.74 (C-2); 135.49 (C-12); 135.75 (C-3); 133.11; 131.05 (C-5); 130.40 (C-19); 129.66 (C-11, C-13); 129.58; 129.32; 129.31 (C-7); 128.48 (C-4); 128.27 (C-22); 127.87; 127.77 (C-6); 114.44 (C-10, C-14); 67.51 (C-8); 19.99 (C-15). FT-IR (ν cm^−1^): 3243w; 2990w; 2927w; 1680m; 1620m; 1578m; 1511vs; 1477m; 1445m; 1385w; 1324s; 1237s; 1191s; 1178m; 1152m; 1054w; 1035m; 1005m; 902w; 857w; 804m; 783w; 758w; 741m; 673m; 604w. Anal. Calcd for C_22_H_17_F_3_N_2_O_2_S (430.44): C, 61.39; H, 3.98; N, 6.51; S, 7.45%; Found: C, 61.57; H, 4.08; N, 6.59; S 7.48%.

*2-((4-Methylphenoxy)methyl)-N-(2,4,5-trifluorophenylcarbamothioyl)benzamide* (**1l**); yield 81%, mp 137.3–138.6 °C. ^1^H-NMR: 12.31 (br s, 1H, NH); 12.11 (br s, 1H, NH); 8.08 (m, 1H, H-22); 7.70 (td, ^3^*J*_(F-18,20-H-19)_= 10.6 Hz, ^4^*J*_(F-21-H-19)_= 7.4 Hz, 1H, H-19); 7.62 (bd, *J =* 7.4 Hz, 1H, H-7); 7.58 (dd, *J =* 1.6 Hz, *J =* 7.4 Hz, 1H, H-4); 7.55 (td, *J =* 1.4 Hz, *J =* 7.4 Hz, 1H, H-5); 7.46 (td, *J =* 1.4 Hz, *J =* 7.5 Hz, 1H, H-6); 7.05 (d, *J =* 8.6 Hz, 2H, H-11, H-13); 6.87 (d, *J =* 8.6 Hz, 2H, H-10, H-14); 5.26 (s, 2H, H-8); 2.21 (s, 3H, H-15). The resonance signal of H-22 is a multiplet, because it is coupled with all three fluorine atoms with different constants, and the ratio between the diference of chemical shifts and coupling constant value is less than 5 (Δν/*J*). ^13^C-NMR: 180.38 (C-16); 170.27 (C-1); 156.01 (C-9); 152.04 (ddd, *J*_(F-18-C-18)_ = 243.8 Hz, *J*_(F-20-C-18)_ = 7.4 Hz, *J*_(F-21-C-18)_ = 2.2 Hz, C-18); 148.02 (ddd, *J*_(F-20-C-20)_ = 248.1 Hz, *J*_(F-21-C-20)_ = 14.7 Hz, *J*_(F-18-C-20)_ = 11.8 Hz, C-20); 145.73 (ddd, *J*_(F-21-C-21)_ = 241.6 Hz, ^2^*J*_(F-20-C-21)_= 13.3 Hz, ^3^*J*_(F-18-C-21)_ = 3.7 Hz, C-21); 135.84 (C-3); 133.02 (C-2); 131.08 (C-5); 129.71 (C-11, C-13); 129.64 (C-12); 128.51 (C-7); 128.40 (C-4); 127.71 (C-6); 122.83 (m, C-17); 115.25 (dd, ^2^*J*_(F-21-C-22)_ = 22.0 Hz, ^3^*J*_(F-20-C-22)_ = 13.2 Hz, C-22); 114.55 (C-10, C-14); 105.93 (dd, ^2^*J*_(F-18-C-19)_ = 21.9 Hz, ^2^*J*_(F-20-C-19)_ = 26.3 Hz, C-19); 67.47 (C-8); 19.96 (C-15). FT-IR (ν cm^−1^): 3249m; 3075w; 2919m; 1676m; 1601w; 1563vs; 1508vs; 1438s; 1384w; 1316s; 1235s; 1215vs; 1204s; 1155s; 1102m; 1054m; 1036m; 942w; 868m; 829w; 804w; 786w; 748w; 703m; 677m; 635w; 585w. Anal. Calcd for C_22_H_17_F_3_N_2_O_2_S (430.44): C, 61.39; H, 3.98; N, 6.51; S, 7.45%; Found: C, 61.71; H, 3.82; N, 6.58; S 7.47%.

*2-((4-Methylphenoxy)methyl)-N-(2,4,6-trifluorophenylcarbamothioyl)benzamide* (**1m**); yield 82%, mp 137.3–138.6 °C. ^1^H-NMR: 12.10 (br s, 1H, NH); 11.56 (br s, 1H, NH); 7.64 (bd, *J =* 7.4 Hz, 1H, H-7); 7.58 (dd, *J =* 1.6 Hz, *J =* 7.4 Hz, 1H, H-4); 7.55 (td, *J =* 1.4 Hz, *J =* 7.4 Hz, 1H, H-5); 7.46 (td, *J =* 1.4 Hz, *J =* 7.5 Hz, 1H, H-6); 7.30 (m, 2H, H-19, H-21, syst. AA’BB’C); 7.07 (d, *J =* 8.6 Hz, 2H, H-11, H-13); 6.88 (d, *J =* 8.6 Hz, 2H, H-10, H-14); 5.26 (s, 2H, H-8); 2.22 (s, 3H, H-15). ^13^C-NMR: 182.69 (C-16); 170.26 (C-1); 160.08 (dt, *J*_(F-C-20)_= 248.2 Hz, ^3^*J*_(F-18,22-C-20)_ = 15.0 Hz, C-20); 159.92 (ddd, *J*_(F-18-C-18)_= 257.8 Hz, *J*_(F-20-C-18)_= 6.6 Hz, *J*_(F-22-C-18)_= 12.3 Hz, C-18, C-22); 156.50 (C-9); 136.39 (C-3); 133.54 (C-2); 130.22 (C-12); 131.62 (C-5); 130.28 (C-11, C-13); 129.20 (C-7); 129.04 (C-4); 128.29 (C-6); 115.24 (C-10, C-14); 101.36 (t, *J*_(F-C)_= 27.8 Hz, C-19, C-21); 68.11 (C-8); 20.60 (C-15). The C-19 and C-21 signal is a triplet due to their scalar coupling with the *orto* fluorine atoms. FT-IR (ν cm^−1^): 3193m; 3071w; 2922w; 1673s; 1644w; 1603w; 1586w; 1527vs; 1509vs; 1447s; 1382w; 1341m; 1289w; 1243s; 1229s; 1174vs; 1122s; 1037s;998w; 853m; 810m; 787w; 753m; 733m; 672m; 509w. Anal. Calcd for C_22_H_17_F_3_N_2_O_2_S (430.44): C, 61.39; H, 3.98; N, 6.51; S, 7.45%; Found: C, 61.74; H, 3.79; N, 6.48; S 7.37%.

### 3.2. Biological Assays

#### 3.2.1. Assessment of the Antimicrobial and Anti-Pathogenic Activity of the Newly Synthesized Compounds 

The *in vitro* qualitative screening of the antimicrobial activity was carried out by an adapted agar-disk diffusion technique using a bacterial suspension of 0.5 McFarland obtained from 24 hour cultures [33,34]. The antimicrobial activities of the newly synthesized compounds were determined against ATCC reference microbial strains, *i.e.*, *S. aureus* ATCC 25923, *Enterococcus faecalis* ATCC 29212, *E. coli* ATCC 25922, as well as clinical strains, recently isolated from different clinical specimens, *i.e.*, *S. aureus* 1694, *Salmonella sp.* 9246, *K. pneumoniae* 1771, *P. aeruginosa* 1671, *C. albicans* 128 and *Aspergillus niger* IC 13534. The microbial strains identification was confirmed by aid of VITEK I automatic system. VITEK cards for identification and susceptibility testing (GNS-522) were inoculated and incubated according to the manufacturer’s recommendations. The results were interpreted by using software version AMS R09.1. The compounds were solubilized in dimethyl sulfoxide to a final concentration of 10 mg/mL. The obtained solutions were further sterilized by filtration. A volume of 10 μL of each tested compounds solution was distributed directly on the solid medium previously seeded with the microbial inocula. The inoculated plates were incubated for 24 hrs at 37 °C. Antimicrobial activity was assessed by measuring the growth inhibition zones diameters expressed in mm [36,37]. The quantitative assay of the minimal inhibitory concentration (MIC, μg/mL) was based on liquid medium two-fold microdilutions and performed in 96 multi-well plates [34,35]. In this purpose, serial binary dilutions of the tested compounds (ranging between 1,000 and 0.975 µg/mL) were performed in a 200 μL volume of nutrient broth/YPG and each well was seeded with 20 μL microbial inocula of 0.5 McFarland density. The plates were incubated for 24 hours at 37 °C for bacterial strains, respectively for 48 hours at 28 °C for fungal strains and MICs were read as the last concentration of compound which inhibited the microbial growth.

#### 3.2.2. Study of the Influence of the Selected Compounds on the Ability of *P. aeruginosa*, *S. aureus* and *K. pneumoniae* to Colonize the Inert Substratum and Form Biofilms

The anti-biofilm activity of the tested compounds was tested by the microtiter method. For this purpose, the microbial strains have been grown in the presence of two-fold serial dilutions of the tested compounds performed in liquid nutrient broth/YPG, distributed in 96-well plates and incubated for 24 hours at 37 °C for bacterial strains, respectively for 48 hours at 28 °C for fungal strains. At the end of the incubation period, the plastic wells were emptied, washed three times with phosphate buffered saline (PBS), fixed with cold methanol and stained with 1% violet crystal solution for 30 minutes. The biofilm formed on plastic wells was resuspended in 30% acetic acid. The intensity of the colored suspensions was assessed by measuring the absorbance at 490 nm. The last concentration of the tested compound that inhibited the development of microbial biofilm on the plastic wells was considered the minimum inhibitory concentration of the biofilm development and was also expressed in μg/mL [36,37].

## 4. Conclusions

New 2-((4-methylphenoxy)methyl)-*N*-(arylcarbamothioyl)benzamides were prepared from 2-(4-methylphenoxymethyl)benzoyl chloride *via* isothiocyanate formation, followed by treatment with various substituted amines. Our results showed that some of the newly synthesized thiourea derivatives carrying aryl groups substituted with one iodine, bromide, fluorine or with two or three chloride atoms could be used for the further development of novel antimicrobial agents with anti-biofilm properties.

## Figures and Tables

**Table 1:** Results of the qualitative screening of the susceptibility spectra of various microbial strains to the newly synthesized compounds (diameter of the growth inhibition zone expressed in mm).

Compound	Microbial strain								
	*Staphylococcus aureus ATCC 25923*	*Staphylococcus aureus 1694*	*Enterococcus faecalis ATCC 29212*	*Pseudomonas aeruginosa 1671*	*Salmonella sp. 9246*	*Klebsiella pneumoniae 1771*	*Escherichia coli ATCC 25922*	*Candida albicans 128*	*Aspergilluss niger IC 13534*
**1a**	0	0	0	0	0	0	0	0	0
**1b**	0	0	0	0	0	0	0	0	0
**1c**	**10**	0	0	0	0	0	0	0	0
**1d**	0	0	0	**6**	0	0	0	0	0
**1e**	0	0	0	0	0	0	0	0	0
**1f**	0	0	0	**12**	0	**10**	0	0	0
**1g**	0	0	0	0	0	0	0	0	0
**1h**	0	0	0	0	0	0	0	0	0
**1i**	0	**11**	0	0	0	0	0	0	0
**1j**	**10**	0	0	**6**	0	0	0	0	0
**1k**	0	0	0	0	0	0	0	0	0
**1l**	0	0	0	0	0	0	0	0	0
**1m**	0	0	0	0	0	0	0	0	0
**DMSO**	0	0	0	0	0	0	0	0	0

**Table 2:** Results of the quantitative assay of MIC for the compounds proving to be active in the qualitative screening on the respective bacterial strains (MIC values expressed in μg/mL).

	Compound				
Strain	1c	1d	1f	1i	1j
***S. aureus* ATCC 25923**	500				500
***S. aureus* 1694**				500	
***K. pneumoniae* 1771**			500		
***P. aeruginosa* 1671**		1000	500		1000

**Table 3:** Results of the quantitative assay of the influence of the tested compounds on the adherence ability and biofilm development on the inert substratum (values of inhibitory concentrations of biofilm development are expressed in μg/mL).

	Compound				
Strain	1c	1d	1f	1i	1j
*S. aureus* ATCC 25923	13				437
*S. aureus* 1694				13	
*K. pneumoniae* 1771			27		
*P. aeruginosa* 1671		13	13		13
